# Annotations

**Published:** 1905-05-06

**Authors:** 


### ANNOTATIONS.

### A Loss to Medical Science.

The death, at the early age of 29, of Dr. Joseph Everett Dutton, is a distinct loss to medical science. Educated at the King's School, Chester, he subsequently graduated at Liverpool University, and in 1897 was appointed to the George Holt Fellowship in Pathology. Dr. Dutton, having entered the Royal Infirmary, Liverpool, where he acted as house surgeon and as house physician, in 1900 made his first expedition to West Africa under the auspices of the Liverpool School of Tropical Medicine in company with Dr. Annett. On their return from Nigeria two reports were issued, one dealing with anti-malarial sanitation and the other consisting of important monographs on Filariasis. After his election in 1901 to the Walter Myers Fellowship, Dr. Dutton proceeded to the Gambia, and drew up a comprehensive anti-malarial report which has proved of the greatest value to the colony. During this expedition he identified in the blood of a patient of Dr. Ford a trypanosome, a parasite which had hitherto been found only in animals; and later on he discovered the same organism in numerous other patients in the Gambia and elsewhere. Enthusiastically pursuing his investigations, he went in 1902 to Senegambia and drew up a report on sanitation which was presented to the French Government. In 1903, in company with Dr. Todd, he went to the Congo to investigate trypanosomiasis and other tropical diseases. They reached Stanley Falls towards the end of last year, and they were able to demonstrate the cause of tick fever in man, and to prove the transference of the disease from man to monkeys. In the pursuit of their researches both of them contracted the malady, but according to a letter from Kosongo, dated February 9th, they had entirely recovered and were returning home in the best of spirits. On Saturday, quite suddenly, came the sad and surprising news that Dr. Dutton had passed away, and that one of the most promising of careers had been cut short. In addition to his remarkable gifts, his unflagging perseverance, and his devotion to the cause of medical science, Dr. Dutton was a young man of great conversational powers, and an altogether charming personality. With the modesty which often accompanies the possession of a brilliant intellect, he always deprecated eulogies of his work, which he said was the source of undiluted pleasure to him. The pity is that the peril which also attached to it has robbed the scientific world of one who had proved himself so well qualified to render pioneer service.

### A Discussion on Rheumatism.

The recently organised Association of Scottish Medical Diplomates has made an encouraging start with its professional work in the shape of a discussion on rheumatism. It was not to be expected that anything particularly novel or startling could be said on this topic, but, judging from the published reports, the discussion was doubtless of interest and value to those taking part in it. Something more than a passing note of agreement and sympathy may be offered to Dr. Morison's eloquent tribute to the work and memory of the late Dr. T. J. Maclagan. Maclagan received but a scanty measure of justice from the profession during his lifetime, and among some recent workers on the subject there appears to be a disposition to forget the insight and courage which marked his introduction of salicin as a remedy for rheumatic fever. Another point of interest raised in the discussion has relation to the prevention of cardiac disease in children who inherit a rheumatic strain. It is allowed that in these children endocarditis is a frequent complication of many ailments which to the lay judgment appear trifling, and which, though grouped by the profession among rheumatic incidents, are not so considered by the public. Hence it is argued that a practitioner who becomes aware in the course of practice of the existence of rheumatic tendencies in any family should instruct the parents in regard to the significance and danger of even apparently trifling illnesses in their children. In this way it is suggested the children would receive the early and complete rest which is recognised as the best means of preventing rheumatic cardiac disease. At present in many instances the rheumatic nature of the illness is only recognised long after the mischief, so far as the heart is concerned, is done. The suggestion is a practical one, and attention might well be paid to it.

### The State and the Birth-Rate.

The decline of the birth-rate in various civilised countries is a subject which has, at occasional intervals during the last few years, attracted public attention. For the most part the comments attached to it have been phrased in lachrymose terms and have formed an introduction to a series of prophecies gloomy with the prospect of national disaster and eclipse. Each nation in turn has served as a whipping boy, and one in particular has been reproached both by the precept and example of its leading citizen. It is hardly uncharitable to suggest that many of these denunciations have been written without any close study of economic law, and that their excuse or opportunity is their adaptation to a readily excited popular emotion. The decline of the family and the ruin of the State are texts which afford much opportunity for eloquence of the flesh-creeping order. At the same time the advocates on the other side hardly betray a more judicious handling of the problem. Their latest suggestion is that the State shall take active steps to control the birth-rate and that a penalty shall be inflicted on those who "overburden society with more than their fair share of offspring." It is hardly a matter for surprise that such a proposal should give pause even to the most enthusiastic adherent of the Malthusian doctrine, and the backers of the new proposal now hasten to explain that their plan is "only a little one." It is to take the form of a "small nominal fine" not as a punishment but as a means of expressing "State censure," and adequate recognition is apparently to be afforded to the "conscientious objector." That any person responsible for such a proposal can seriously believe himself competent to deal in a practical spirit with so difficult and delicate a matter as the population question is an example of self-delusion not easily equalled.

